# Coal-based 3D hierarchical porous carbon aerogels for high performance and super-long life supercapacitors

**DOI:** 10.1038/s41598-020-64020-5

**Published:** 2020-04-27

**Authors:** Yan Lv, Lili Ding, Xueyan Wu, Nannan Guo, Jixi Guo, Shengchao Hou, Fenglian Tong, Dianzeng Jia, Hongbo Zhang

**Affiliations:** Key Laboratory of Energy Materials Chemistry, Ministry of Education; Key Laboratory of Advanced Functional Materials, Autonomous Region; Institute of Applied Chemistry, Xinjiang University Urumqi, Urumqi, 830046 P. R. China

**Keywords:** Chemistry, Energy science and technology, Materials science

## Abstract

Coal-based 3D hierarchical porous carbon aerogels (3D HPCAs) has been successfully fabricated from a freeze-drying method and with subsequent of calcination process, using coal oxide as carbon precursors, and PVA as both cross-linking agent and sacrifice template. The 3D HPCAs, using as electrode materials for supercapacitors, display outstanding electrochemical performance. The optimal sample (HPCAs-0.4-800) presents a high specific capacitance of 260 F g^−1^ at 1 A g^−1^, and exhibits considerable rate capability with the retention of 81% at 10 A g^−1^. Notably, HPCAs-0.4-800 shows an excellent cycling stability with 105% of the capacitance retention after 50000 cycles at 10 A g^−1^, attributing to its unique hierarchical porosity, high surface area up to 1303 m^2^ g^−1^, and improved conductivity. This work offers a promising route to synthesize coal-based porous carbon aerogels electrode materials for supercapacitors.

## Introduction

Supercapacitors (SCs), also called ultracapacitors or electrochemical capacitors, have caused a large amount of interest owing to excellent electrochemical stability, fast charge/discharge, high power density and environmental friendly^1–5^. Supercapacitors store electrical charge on high-surface-area conductive materials, so its performance mainly relies on the electrode materials. Outstanding electrode materials should possess ion approachable high surface areas for high specific capacitance and fasted electron transfer for excellent rate capacity^6,7^. So it is very crucial for supercapacitors with high performance to prepare electrode materials with proper architecture structure, suitable pore size distribution and high specific surface area (SSA)^8^. Among the numerous electrode materials of supercapacitors, carbon materials have attracted more attention because of their unique physical and chemical properties^9,10^. Carbon aerogels (CAs), as one of carbon materials, show outstanding characteristics, such as low density, developed porosity, and multi-branched network structure^11–13^. These structural features can afford the quick transfer channel for ion migration and more active sites, which can lead to the excellent electric double layer performance in supercapacitors. To improve the specific surface area and porosity, most of the CAs are prepared by using pore-forming agents, such as strong bases^14–16^, hard templates^17–19^, soluble salts^20,21^, soft templates and so on^22–24^. Among of them, the soft templates can be directly decomposed during the carbonization process instead of etching procedure using harmful and toxic or corrosive chemicals. Therefore, it has been attracting extensive attention to prepare CAs using soft template for the application of supercapacitors.

Currently, the researches of CAs are mainly focused on precursors, such as resorcinol-formaldehyde^25^, polymers^26^, nanotubes^27,28^, graphene^29,30^, and natural precursors such as cellulose and glucose^31,32^. In our previous works, we have fabricated some functional materials on coal of traditional fossil, such as porous spheres^33^, fibers^34^, bamboo-like carbon nanotubes (CNTs)^35^, graphene quantum dots (GQDs)^36^ and hierarchical porous carbon^37^. All of them demonstrate that the coal can be used to fabricate functional carbon materials. However, so far, coal-based porous carbon aerogels have been few reported and the preparation processes were very complicated and the yield was low in a few studies. Therefore, it is still a great challenge to design simple and productive approaches for the controllable synthesis coal-based porous carbon aerogels.

In our work, we developed an efficient method to construct coal-based 3D HPCAs by carbonization of freezing-dried PVA/coal-based hydrogels, in which coal oxide serves as the carbon source and PVA serves as the sacrificial template and cross-linking agent, respectively. The amount of mesoporous and micropores of the 3D HPCAs can be controlled by tuning the mass ratio of coal oxide and PVA. The performance of the obtained 3D HPCAs are evaluated as the electrode materials of supercapacitors. The optimal sample displays an excellent electrochemical performance. It exhibits a specific capacitance up to 260 F g^−1^ in the three-electrode system at 1 A g^−1^, and a high rate performance of 187 F g^−1^ at 20 A g^−1^, as well as a remarkable cycling stability (105% of capacitance retention after 50000 cycles). More importantly, the specific capacitance measured was 201.1 F g^−1^ at the current density 1 A g^−1^ in an assembled symmetrical cell system, and good cycling stability with 108% over 10000 cycles at 4 A g^−1^. The excellent electrochemical performance may be attributed to the characteristic of 3D cross-linked structure with SSA up to 1303 m^2^ g^−1^, hierarchical porous structure and appropriate ratio of micropore volume to total volume of 65.6%. The materials with hierarchical porous structure can be used as potential electrode materials for energy conversion and storage, and this work provides a green way for high-value utilization of coal in energy storage.

## Results and discussion

**Figure 1:**
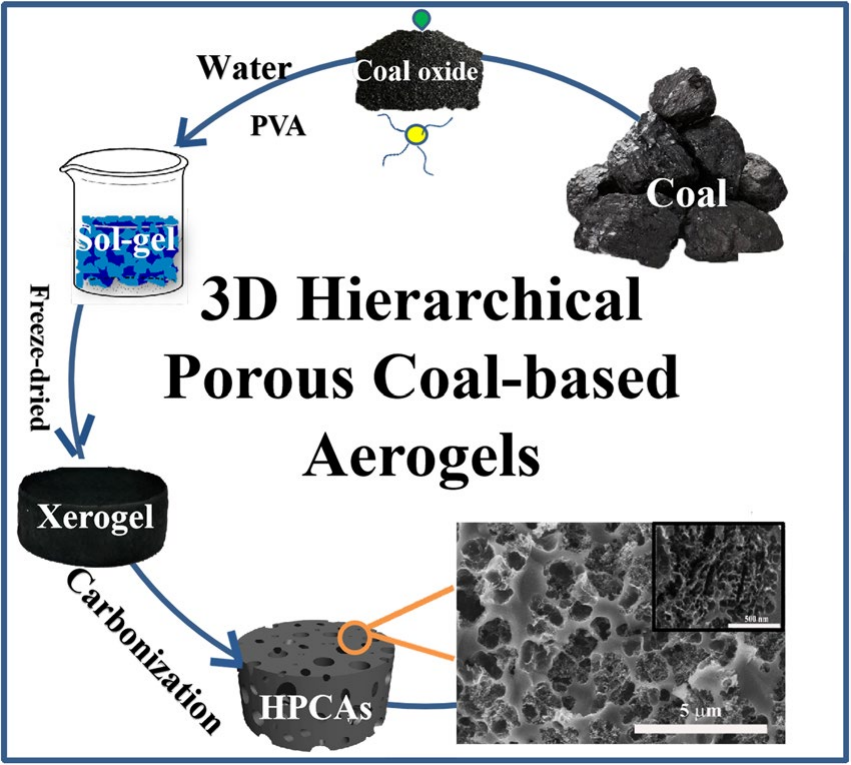
The synthetic scheme of 3D HPCA. (drawn by Microsoft PowerPoint 2010 and 3D Studio Max 8 software).

The synthesis procedure of the 3D HPCAs is illustrated in Fig. 1. Firstly, PVA/coal-based hydrogels were prepared by using PVA as a crosslinking agent of coal oxide fragment, and then the 3D network porous structures were formed through a freeze-drying and with subsequent of calcination. To understand the role of PVA and coal oxide in 3D HPCAs, thermogravimetric (TG) analyses of PVA and coal oxide were studied (Fig. S1). The mass loss of PVA is 96.6% of initial weight from 260 °C to 490 °C, and 98.8% when heated to 800 °C, while that of coal oxide is about 62.6% when heated up to 800 °C under an argon atmosphere. The results demonstrate that coal oxide is the primary carbon source in the 3D HPCAs, while PVA is the cross-linking agent for formation of hydrogels and the sacrifice template to fabricate 3D network porous structures.

**Figure 2:**
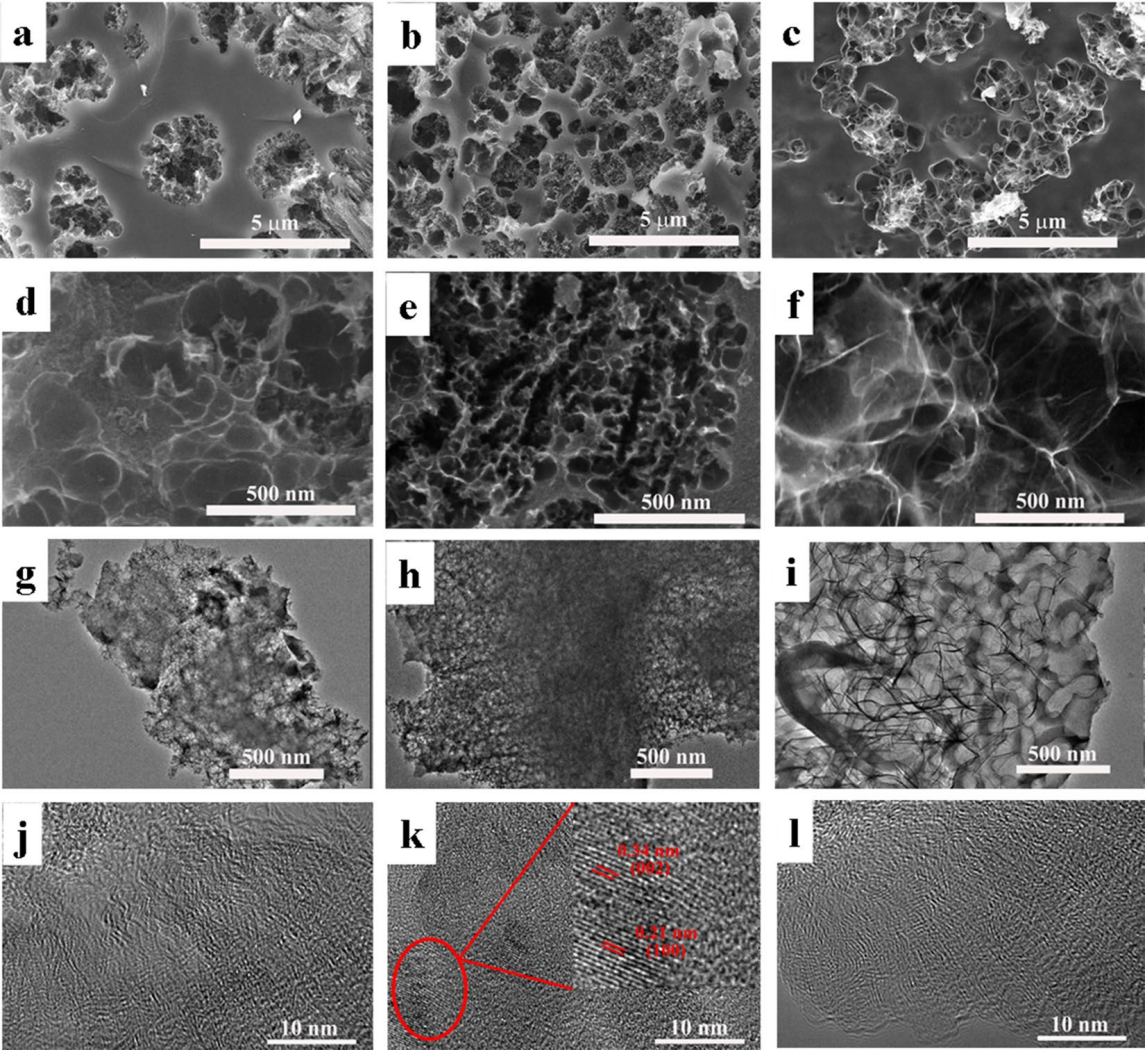
SEM images of 3D HPCAs (**a**,**d**) HPCAs-0.2-800; (**b**,**e**) HPCAs-0.4-800; (**c**,**f**) HPCAs-0.8-800; TEM images of HPCAs (**g**) HPCAs-0.2-800; (**h**) HPCAs-0.4-800; (**i**) HPCAs-0.8-800; HRTEM images of HPCAs (**j**) HPCAs-0.2-800; (**k**) HPCAs-0.4-800; (**l**) HPCAs-0.8-800.

This result is also directly proved by the optical and SEM images of the pure oxidized coal-800, prepared by the same proceduce only without addition of PVA. As shown in Fig. S2a,b, the pure oxidized coal-800 shows powder shape but non-aerogel on the macro level, and block shape on the micro level, with dense surface and no obvious macropores and mesopores. The morphology of fabricated 3D HPCAs were directly observed by scanning electron microscopy (SEM) and transmission electron microscopy (TEM) (Fig. 2). The SEM images show that the pore structure of 3D HPCAs changed obviously as the increase of PVA content. Consistent well with SEM images, the TEM images of HPCAs-0.4-800 display a richer porous structure and more even pore size distribution obviously. The results demonstrate that an appropriate amount of PVA is crucial for the formation of the pore structure of 3D HPCAs. Coal oxide will stack seriously when crosslinking dose of PVA is excessive, on the contrary, too little sacrificial dose of PVA is not conducive for the formation of holes during the carbonization process. In addition, the influence of calcination temperature on the pore structure of 3D HPCAs was also studied. As shown in Fig. S3, the pore size of HPCAs-0.4-700 and HPCAs-0.4-900 increase due to the change of carbonized temperatures. The broken mesopores can be observed in the HPCAs-0.4-900 due to the skeleton collapse at high carbonization temperature.

**Figure 3:**
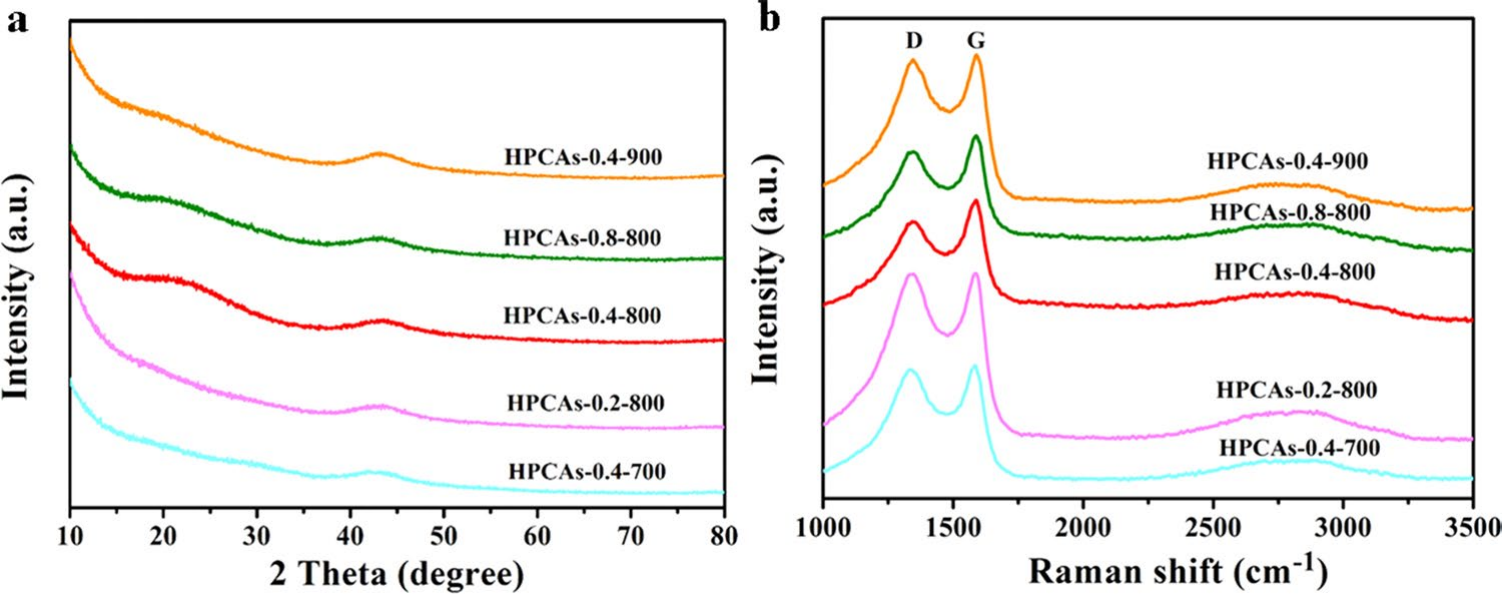
(**a**) XRD patterns and (**b**) Raman spectra of 3D HPCAs.

The effect of different components and calcination temperatures on the structure of the HPCAs was investigated by X-ray powder diffraction (XRD) (Fig. 3). XRD patterns of 3D HPCAs all display two weak peaks located at 24° and 43°, associated with diffraction of the (002) and (101) planes carbon. Compare with the peaks of coal oxide (Fig. S4), the peaks around 23° of the HPCAs shift up to a relatively high angle, indicating that the degree of graphitization is reduced during the process of carbonization^39–41^. In addition, the HPCAs-0.4-800 displays the greatest peak intensity at 23° among of all HPCAs, indicating that the HPCAs-0.4-800 has the highest degree of graphitization associated with conductivity. Raman spectra are shown in Fig. 3b and S3, two obvious peaks around 1350 and 1590 cm^−1^ are attributed to D and G bands. The D peak is attributed to the sp^3^ defects of disordered ones in the hexagonal graphitic layers and sp^2^ carbon with O-containing groups and H-sites, as well as domain boundary. The G peak reflects the vibration of sp^2^-bonded carbon atoms^42–44^. The I_D_/I_G_ values of HPCAs-0.4-700, HPCAs-0.2-800, HPCAs-0.4-800, HPCAs-0.8-800, HPCAs-0.4-900 and coal oxide are 0.99, 0.97, 0.87, 0.90, 0.97 and 0.81 respectively. Compared HPCAs with coal oxide (Fig. S4), the HPCAs show higher values of I_D_/I_G_, because the surface carbon atoms of HPCAs were carried off during the activation operation, thus, leaving the free bond at the surface and forming a disordered carbon structure^1,45,46^. In addition, the HPCAs-0.4-800 has the lowest I_D_/I_G_ value in the samples of different calcination temperatures, which likely resulted from the reduction reaction at high temperature, on the other hand, the defects of materials increase as the structure collapse at a too high carbonization temperature (900 °C). And among the samples of HPCAs-0.2-800, HPCAs-0.4-800, and HPCAs-0.8-800, HPCAs-0.4-800 also exhibits the highest graphitization degree, which acts as a key role in improving the conductivity. This is because excessive PVA decomposition at high temperatures causes the pore structure to collapse, thus forming an amorphous carbon. The above results prove that appropriate carbonization temperature and PVA content are crucial to the pore structure and the degree of graphitization, which finally reflected in its electrochemical performance.

The XPS spectra were carried out to evaluate the surface atomic composition of HPCAs-0.4-800. Fig. S5 shows the XPS spectra of samples. The survey spectrum confirmed the existence of C, O, and N elements in the sample of HPCAs-0.4-800 (Fig. S5a). The results are consistent with the FT-IR spectra data analysis reported in our previous work^38^. The N and O in samples are mainly come from the raw coal and nitric acid and sulfuric acid used in the oxidation process. The high-resolution spectrum of C 1s (Fig. S5b) can be divided into three peaks at 288.6, 286.2 and 284.5 eV, which are assigned to C=O, C-O and C-C, respectively^47,48^. The O 1s spectrum (Fig. S5c) consists of four peaks located at 530.9, 531.5, 532.4 and 533.5 eV, which corresponding to the carbonyl groups (C=O), bridge-bonded oxygen (C-O-C), ester groups (O-C=O) and chemisorbed oxygen or water (COOH carboxylic groups or water), respectively. The high resolution spectrum of N 1s (Fig. S5d) shows three peaks with binding energy values of 398.1, 400.5 and 404.3 eV for the pyridinic-N, pyrrolic-N and oxidized-N, respectively^49,50^. The elemental contents (atomic%) of the HPCAs-0.4-800 obtained from XPS data are presented in Table S1. HPCAs-0.4-800 has high contents of oxygen and nitrogen, with a ratio of 7.27% and 1.6%, respectively, which can ameliorate the wettability between the electrode material surface and electrolytes, further facilitate the immersion of electrolyte into the interior of the electrode materials, and ultimately reflect in high capacitance performance. Besides, the pyridinic-N and pyrrolic-N can introduce the faradaic pseudocapacitance in aqueous electrolytes and thus also enhance electrochemistry capacitance^51^.

**Figure 4:**
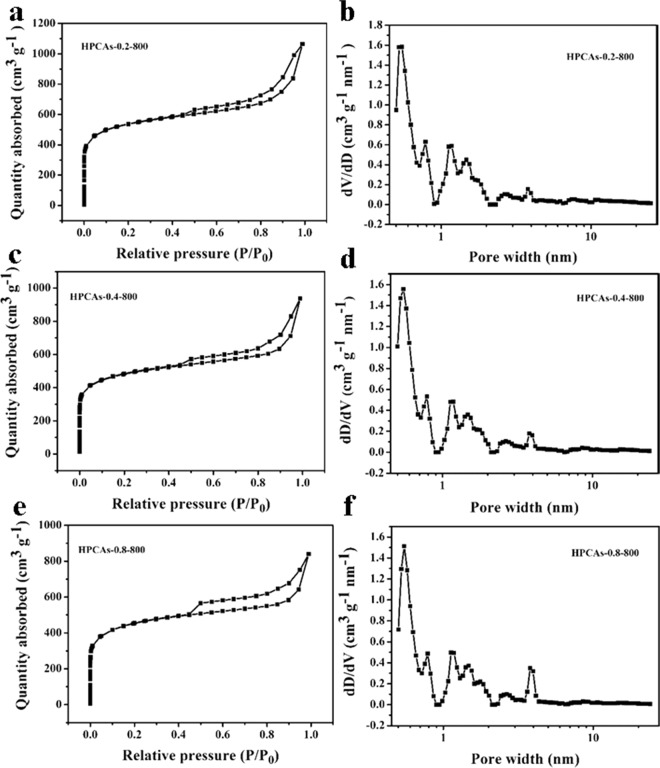
N_2_ adsorption-desorption isotherms (**a**,**c**,**e**) and pore size distributions (**b,d,f)** of HPCAs-0.2-800, HPCAs-0.4-800 and HPCAs-0.8-800.

The electric double layer capacitor (EDLC) is a surface regulated phenomenon, so a larger surface area is crucial for acquiring a high capacitance^52^. The N_2_ adsorption-desorption isotherms and the pore distribution of samples with the different mass ratios of coal oxide/PVA are presented in the Fig. 4a–f. The isotherms of the 3D HPCAs show classical type-IV curves with an H4 type hysteresis loop in the relative pressure region between 0.45 and 1.0, suggesting that the existence of a silt-shaped pore structure. Compared with the pure oxidized coal-800 (Fig. S2c), the volume of adsorbed N_2_ increase steeply at relatively low pressure, which stands for the existence of a large number of micropores, and the remarkable hysteresis loop between N_2_ adsorption and desorption branch manifests the existence of mesopores. It is well known that micropores and mesopores are in favor of the improvement of charge storage and ion transport, respectively^53^. DFT pore-size-distribution curves show that the HPCAs have broad micropores size distribution (0.65-2 nm) and a narrow mesopores size distribution (2-10 nm). The sizes of micropores are close to the size of hydrolyzed K^+^ ions (0.331 nm), which are beneficial for their capacitive performance^54^. The data of S_BET_ and pore distribution of 3D HPCAs are shown in Table 1. S_BET_ of HPCAs-0.2-800, HPCAs-0.4-800, HPCAs-0.8-800 are 1018, 1303, and 847 m^2^ g^−1^, respectively. Distinctly, the contribution of micropores to S_BET_ of HPCAs-0.4-800 (66%) was greatest among all samples (44% to HPCAs-0.2-800 and 55% to HPCAs-0.8-800). In general, the large specific surface area of micropores can result in a high capacity^6,55^. The N_2_ adsorption-desorption isotherms and the pore distribution of samples with different calcination temperatures (HPCAs-0.4-700 and HPCAs-0.4-900) are shown in Fig. S6. They show similar types of type-IV curves with HPCAs-0.4-800. The effects of the calcination temperature on distribution of hierarchical porous have also been studied, as shown in Table S2. The S_BET_ of HPCAs was 971, 1303, and 900 m^2^ g^−1^ at 700, 800, and 900 °C, respectively. The contribution of micropores to S_BET_ of HPCAs-0.4-800 (66%) was also larger than that of HPCAs-0.4-700 (54%) and HPCAs-0.4-900 (46%). This is because the high temperature promotes the decomposition of the large amounts of coal oxide and PVA, leading to achieve abundant pores, but too high a temperature (900 °C) will cause the stacking of carbon layers and/or generation of isolated pores, reflecting a smaller S_BET_ and the amount of micropores. Such result is related to their electrochemical performances.

**Table 1:** BET Specific surface area and porous structure of HPCAs-0.2-800, HPCAs-0.4-800 and HPCAs-0.8-800.

Sample	S_BET_^*a*^ (m^2^ g^−1^)	V_total_^b^ (cm^−3^ g^−1^)	V_meso_^c^ (cm^−3^ g^−1^)	S_micro_^d^ (m^2^ g^−1^)	D_ap_^e^ (nm)
HPCAs-0.2-800	1018	1.65	0.78	450	6.4
HPCAs-0.4-800	1303	1.45	0.61	856	4.4
HPCAs-0.8-800	847	1.30	0.80	427	6.1

^a^BET surface area. ^b^The total pore volume at *P/P*_*o*_ = 0.99, ^c^The mesopore volume calculated using the BJH method based on the Kelvin equation. ^d^Micropore surface area calculated using the V-t plot method. ^e^Average pore size (4V_t_/S_BET_).

**Figure 5:**
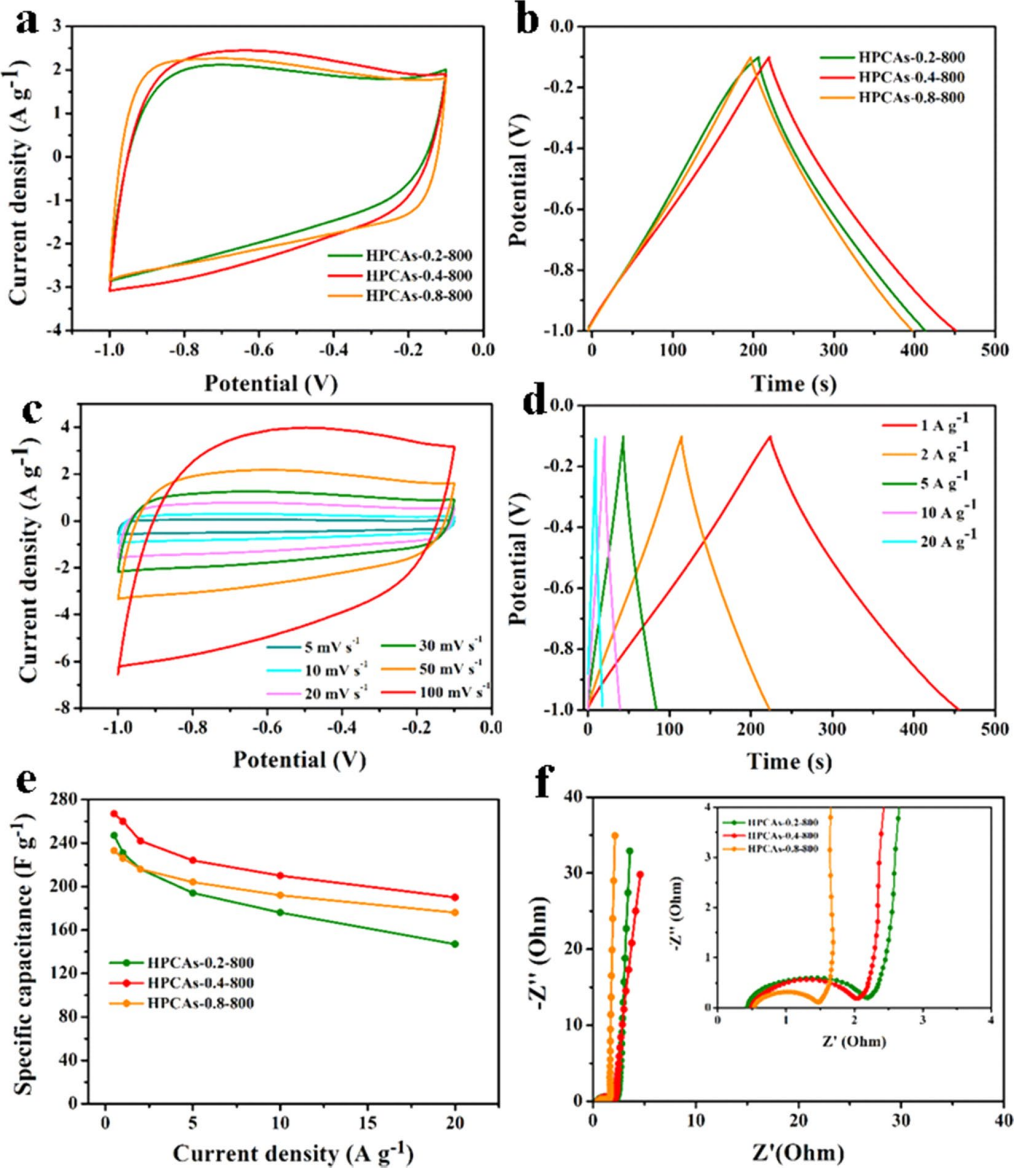
(**a**) CV curves at the scan rate of 50 mV s^−1^ and (**b**) GCD cures at the same scan rate of 1 A g^−1^ of the samples with different PVA content; (**c**) CV curves and (**d**) GCD cures of the HPCAs-0.4-800 sample at different current densities; (**e**) Specific capacitances of the samples with different PVA content at the current density of 0.5-20 A g^−1^; (**f**) Electrochemical impedance spectra of 3D HPCAs as Nyquist plots.

The electrochemical performances of 3D HPCAs were evaluated through CV curves at the scan rate of 50 mV s^−1^ and GCD curves at the current density of 1 A g^−1^ (Fig. 5a,b). All 3D HPCAs electrode materials display similar rectangles, suggesting that the energy storage type of the 3D HPCAs are the electric double layer capacitor (EDLC). The values of specific capacitance are 232, 260, 218, 210 and 184 F g^−1^ at the current density of 1 A g^−1^ for HPCAs-0.2-800, HPCAs-0.4-800, HPCAs-0.8-800, HPCAs-0.4-700 and HPCAs-0.4-900, respectively, which is all are superior to that of pure oxidized coal-800 (35.7 F g^−1^ Fig. S2e). The 3D HPCAs-0.4-800 exhibits the highest specific capacitance among all the electrode materials due to advantages of the large accessible surface area, more available mesoporous channels and advisable proportion of micropore volume to total volume. As shown in the Fig. 5c,d, specific capacitances of HPCAs-0.4-800 obtained from the discharge curve are 267, 260, 242, 224 and 210 F g^−1^ at the current density of 0.5, 1, 2, 5 and 10 A g^−1^. On account of the insufficient surface contact and hindrance of ions diffusing into the internal pores, the capacitance decreases as the current density increased^56^. The calculated specific capacitances of the HPCAs at different current densities are presented in Fig. 5e. The 3D HPCAs-0.4-800 electrode material displays the specific capacitance of 260 F g^−1^ at 1 A g^−1^, which is higher than HPCAs-0.2-800, HPCAs-0.8-800, and the commercial activated carbon (Kuraray YF-50, 87 F g^−1^) (Fig. S7). Such result is probably ascribed to that sufficient micropores and mesopores channel to be used. The specific capacitance of HPCAs-0.4-800 is 210 F g^−1^ at 10 A g^−1^, which is about 80.7% of the capacitance retention of 260 F g^−1^ at 1 A g^−1^. However, when the current density increases from 1 A g^−1^ to 10 A g^−1^, HPCAs-0.2-800 and HPCAs-0.8-800 have the only 74.8% and 78.9% capacitance retention, respectively. HPCAs-0.4-800 manifests a considerably better rate performance than HPCAs-0.2-800 and HPCAs-0.8-800 samples, because of HPCAs-0.4-800 have uniformed and well-interconnected hierarchical porous structure. On the other hands, compared to the samples of HPCAs-0.4-700 and HPCAs-0.4-900, HPCAs-0.4-800 has the highest SSA and the suitable ratio of micropore volume to total volume of 65.6%, which is good for charge storage. Therefore, it has the highest specific capacitance.

Electrochemical impedance spectroscopy (EIS) were measured to compare the electrochemical kinetics of the samples. Nyquist plots of HPCAs electrode materials consist of a vertical line and a semicircle at the low frequency and the high frequency area, respectively (Fig. 5f, and S8c). The intercept at the real axis of high frequency of all electrodes are nearly the same, indicating their similar ohmic resistance (R_s_) of 0.5 Ω. A smaller semicircle at the high frequency, reflects a relatively lower charger transfer resistance (R_ct_)^57^. The R_ct_ values of HPCAs are 3.1, 2.5, 1.8, 1.5, and 1.1 Ω for HPCAs-0.4-900, HPCAs-0.4-700, HPCAs-0.2-800, HPCAs-0.4-800 and HPCAs-0.8-800, respectively. The HPCAs-0.4-800 has the smallest R_ct_ among three samples of different carbonization temperature. The result is due to that HPCAs-0.4-800 has the highest degree of graphitization and comparatively abundant pore structure. Besides, the R_ct_ decreased with the increase of the PVA content in samples, which is likely attributed to more available mesoporous channels and more N content coming from PVA. On the other hand, all samples show nearly perpendicular to the imaginary axis in the low frequency region, which indicated that the electrolyte ions had the best diffusion ability in electrode structure. And the straight line demonstrates the ideal EDLC behavior of electrode materials^58,59^.

From the above results, the HPCAs-0.4-800 exhibits the excellent electrochemical property. The specific capacitance performance of HPCAs-0.4-800 is superior to some previously reported porous carbon materials (Table S3). As mentioned above, the excellent capacitive performance of HPCAs-0.4-800 can be ascribed to the following aspects: (i) the carbon yields of coal oxide and PVA are different, which leads to the formation of hierarchical porous materials. (ii) affluent mesopores provide adequate ion transfer passageway. (iii) the unique 3D hierarchical porous structure ensures efficient contact with the electrolyte.

**Figure 6:**
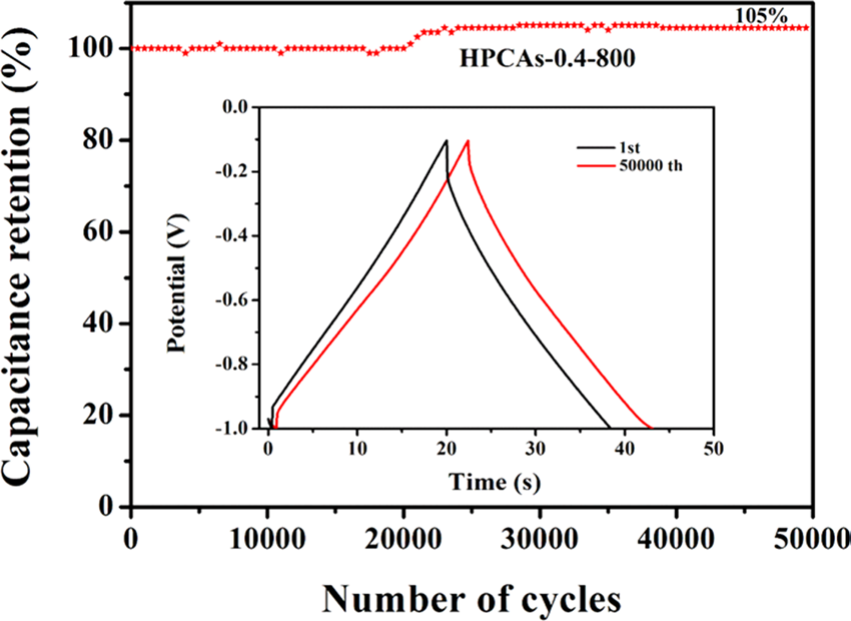
Cycling performance of the HPCAs-0.4-800 electrode at 10 A g^−1^ (the inset displays GCD cures of the HPCAs-0.4-800 electrode at 1 and after 50000 discharge/charge cycles at 10 A g^−1^).

The stable cycling life is an important factor for the practice application of supercapacitor electrode materials. Fig. 6 shows that the specific capacitance of the HPCAs-0.4-800 still reaches 230 F g^−1^ at the high density of 10 A g^−1^, and superior capacitance is maintained up to 105% after 50000 cycles. What impressed us most is that the capacitance presents an increasing trend during the repeating process of cycling at the high current density of 10 A g^−1^. On the basis of the pore distribution, this phenomenon has to do with the porous structure of the electrode materials. To be specific, in the beginning stages, only large pores and mesopores are infiltrated by electrolyte, the micropore structure is not fully utilized due to the thin film on the electrode of supercapacitors^60^. However, the K^+^ hydrated ions can gradually penetrate into the micropores and participate in the establishment of electric double layers^61^. The electrode material of 3D HPCAs-0.4-800 tends to a stable capacitance value due to the full utilization of pores as the cycles increasing. Therefore, the sample has a considerable reversibility and satisfactory cycle stability during the repeated charge-discharge process.

**Figure 7:**
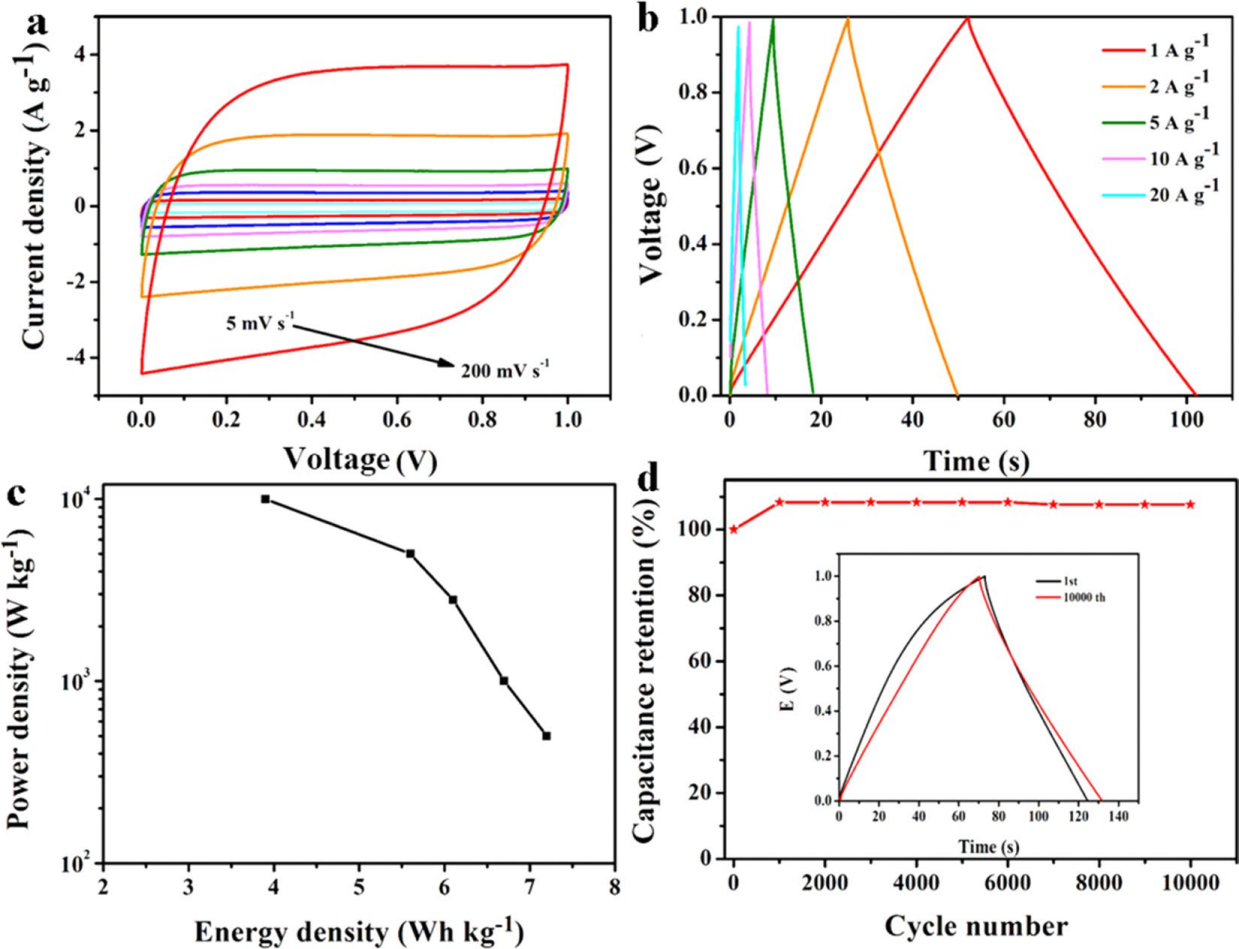
Electrochemical performances of the HPCAs-0.4-800//HPCAs-0.4-800 symmetric cell: (**a**) CV curves at various sweep rates. (**b**) GCD curves recorded at 1-20 A g^−1^. (**c**) Ragone plot. (**d**) Cycling stability measured at 4 A g^−1^ with inset showing GCD curves (1 A g^−1^) before and after 10,000 cycles.

Simultaneously, the electrochemical performance of the HPCAs-0.4-800 in symmetric cell system was also investigated. Fig. 7a shows that the HPCAs-0.4-800 has rectangular CV curves at different scan rates, indicating distinct capacitive behavior and fine reversibility. Fig. 7b shows the galvanostatic charge-discharge (GCD) curves at different current densities from 1 to 20 A g^−1^ in the potential range from 0 to 1 V. The HPCAs-0.4-800 shows an excellent specific capacitance of 201.1 F g^−1^ at 1 A g^−1^ and 160.0 F g^−1^ at 20 A g^−1^, respectively. And the electrode has a good rate capability with about 80% capactive retention at 20 A g^−1^. As shown in Fig. 7c, the HPCAs-0.4-800-based device has a pretty high energy density (7.2 Wh kg^−1^ at 500 W kg^−1^). Fig. 7d manifests the cycling stability of the HPCAs-0.4-800//HPCAs-0.4-800 cell. The specific capacitance retention up to 108% after 10,000 cycles due to the fully wetting of sufficient pores during the long time charged/discharged process, exhibiting its outstanding stable cycling. To sum up, the excellent electrochemical performances of HPCAs-0.4-800, such as high specific capacitance, and good cycling stability can be attributed to the 3D hierarchical porous and the appropriate microspores and mesopores size distribution as well as improved conductivity.

## Conclusion

In summary, we design a low cost and facile strategy to obtain coal-based 3D HPCAs by carbonization of the freeze dried PVA/coal-based hydrogels. The structure and electrical performances of 3D HPCAs are adjusted and optimized by changing the content of PVA and carbonization temperature. Among of all samples, the HPCAs-0.4-800, as an electrode material of supercapacitors, exhibits excellent specific capacitances of 260 F g^−1^ and 201.1 F g^−1^ at 1 A g^−1^ in 6.0 M KOH electrolyte for the three- and two-electrode systems, respectively. It also displays an excellent cycling stability of 105% capacitance retention after 50000 cycles. This work provides a promising route to construct coal-based 3D HPCAs as highly efficient electrodes materials for supercapacitors.

## Experiment Section

### Materials

Coal was obtained from Heishan, Xinjiang, China. The similar analysis of coal has been reported by our group^38^. Polyvinyl alcohol (PVA) (molecular weight is 44.05 MW) purchased from Sigma-Aldrich. H_2_SO_4_ (98%), HNO_3_ (63%), KOH were analytical grade.

### The preparation of 3D HPCA

Coal oxide was firstly obtained by treating raw coal using a mixed acid (VHNO_3_/VH_2_SO_4_ = 1:3) as previously reported by our group^38^. Then PVA/coal-based hydrogels were synthesized as following processes. Firstly, coal oxide (0.8 g), PVA (0.4 g) were dissolved in 10.0 mL deionized water and the pH of the solution was neutralized with the KOH, and then stirred at 80 °C continuously for 12 h. Secondly, the product was frozen in refrigerator (−70 °C) for 24 h and freeze-dried in vacuum for 24 h to obtain the xerogels. Finally, the as prepared xerogel was calcined at 800 °C for 2 h with a heating rate of 5 °C min^−1^ under flowing N_2_ atmosphere for formation of 3D coal-based HPCAs, the sample was called HPCAs-0.4-800. Similarly, HPCAs-0.2-800 and HPCAs-0.8-800 were prepared by the nearly same methods with the only different mass ratios of coal oxide/PVA (0.8/0.2, 0.8/0.8). For the two others of coal based HPCAs annealed at 700 °C (denoted as HPCAs-0.4-700) and 900 °C (denoted as HPCAs-0.8-900) were synthesized for comparison. And the pure oxidized coal-800, as a control sample, was prepared by the same methods only without addition of PVA and annealed at 800 °C.

### Structural characterization

SEM and TEM images were recorded on field emission scanning electron microscopy (FESEM Hitachi SU-4800) and transmission electron microscopy (TEM, JEM-2100F), respectively. Thermogravimetric analysis (TGA) was tested by using a NETZSCH STA449F3-QMS403C instrument under N_2._ XRD measurements were carried out on an X-ray diffractometer (XRD, Bruker D8, using filtered Cu Kα radiation). X-ray photoelectron spectroscopy (XPS) spectra and the Raman spectrum were recorded with a Thermo ESCALAB 250 instrument (Al Ka X-ray source) and a Bruker Senterra spectrometer (532 nm) Raman spectrometer, respectively. SSA and pore size distribution of coal-based 3D HPCAs were determined on Autosorb-IQ, Quantachrome by BET method.

### Electrochemical characterization

The electrochemical experiments of coal-based 3D HPCAs were tested in the three-electrode system in 6.0 M KOH electrolyte at room temperature, in which the 3D HPCAs use as working electrode, Ag/AgCl and Pt foil (1 cm^2^) as the reference electrode and counter electrode, respectively. The working electrodes were prepared by mixing 3D HPCAs, carbon black and [Poly (trafluoroethylene)] at a mass ratio of 8:1:1 in absolute ethyl alcohol (the active mass is about 2.0 mg), and then the mixture was pasted on the nickel form, and dried at 60 °C for 12 h. The electrochemical performances of these electrodes were carried out using the CHI 660D electrochemical workstation (Chenhua, China). The current density of 10 A g^−1^ was applied in cyclic GCD measurements for over 50,000 cycles (the potential is −1.0 – −0.1 V). The specific capacitance (C_S_) of the 3D HPCAs electrode materials were calculated via Eq. (1).

1 $${{\rm{C}}}_{{\rm{S}}}={\rm{I}}\times \Delta {\rm{t}}/({\rm{m}}\times \Delta {\rm{V}})$$

Where I, ∆t, ∆V, m is current, discharging time, potential window and the mass of the active material, respectively.

In two-electrode system, CV and GCD curves were measured using the product in 6.0 M KOH as the electrode (the total mass of the active on two working electrodes is 4.0 mg). The specific capacitance for the single electrode (C_sp_) was obtained via Eq. (2).

2 $${{\rm{C}}}_{{\rm{sp}}}=4{\rm{I}}\times \Delta {\rm{t}}/({\rm{M}}\times \Delta {\rm{V}})$$

Where, ∆V refers to the voltage range, M represents the total mass of the active materials on two working electrodes, ∆t is the discharge time, I is the discharge current.

3 $${\rm{E}}={{\rm{C}}}_{{\rm{sp}}}\Delta {{\rm{V}}}^{2}/(8\times 3.6)$$

4 $${\rm{P}}=3600\times {\rm{E}}/\Delta {\rm{t}}$$

Where E, P are the energy density (Wh kg^−1^), power density (W kg^−1^), respectively.

## Supplementary information

Supplementary information.
