# Supplementary material for: Coal-based 3D hierarchical porous carbon aerogels for high performance and super-long life supercapacitors
Source: Sci Rep. 2020 Apr 27;10:7022. doi: 10.1038/s41598-020-64020-5 (PMC7184571; doi:10.1038/s41598-020-64020-5)
Supplement: Supplementary file 1 — Supplementary information. [file 41598_2020_64020_MOESM1_ESM.pdf]

Coal-based 3D hierarchical porous carbon aerogels for high performance and super-long life supercapacitors

Yan Lv, Lili Ding, Xueyan Wu, Nannan Guo, Jixi Guo,* Shengchao Hou, Fenglian Tong, Dianzeng Jia*, Hongbo Zhang

Key Laboratory of Energy Materials Chemistry, Ministry of Education; Key Laboratory of Advanced Functional Materials, Autonomous Region; Institute of Applied Chemistry, Xinjiang University Urumqi, 830046 China
E-mail: jxguo1012@163.com; jdz@xju.edu.cn

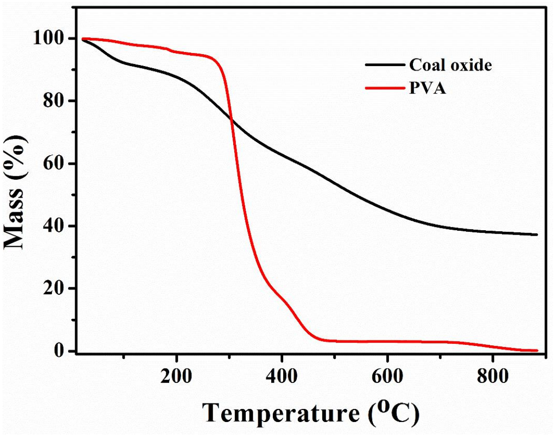

Figure S1. TG curves of coal oxide and PVA.

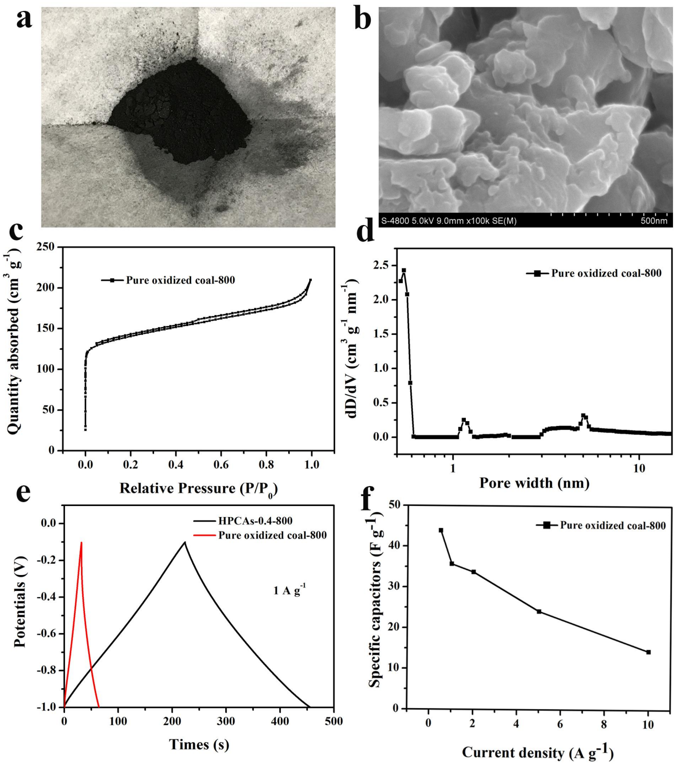

Figure S2. (a,b)The optical and SEM images, (c,d)N_2 adsorption-desorption isotherms and pore size distributions (e,f) the electrochemical performances of the pure oxidized coal-800.

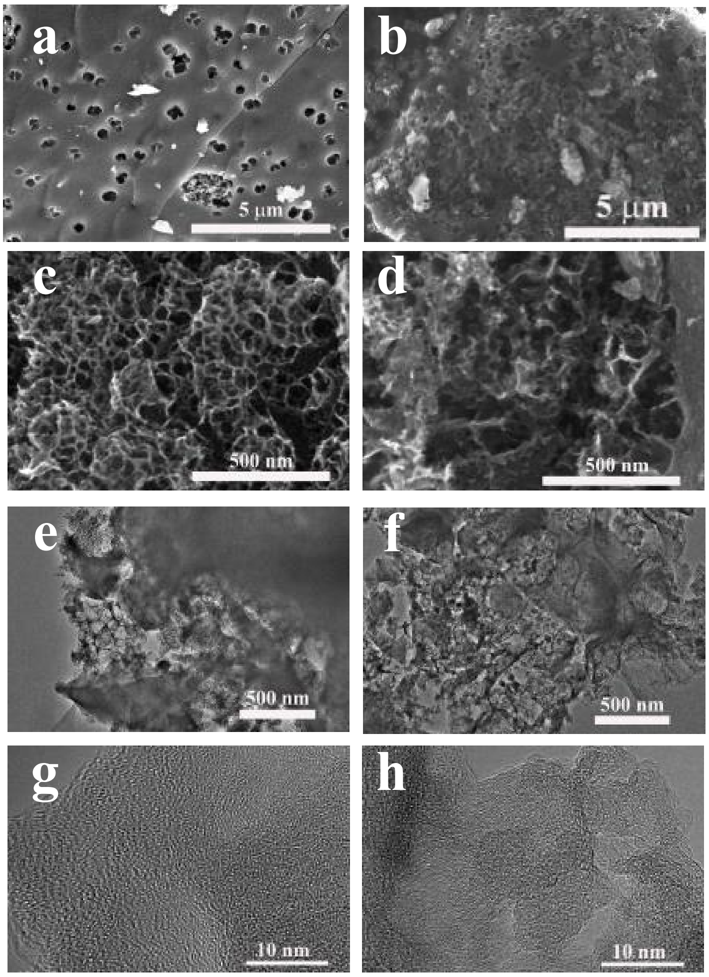

Figure S3. SEM images of HPCAs (a, c) HPCAs-0.4-700; (b, d) HPCAs-0.4-900; TEM images of HPCAs (e) HPCAs-0.4-700; (f) HPCAs-0.4-900; HRTEM images of HPCAs (g) HPCAs-0.4-700; (h) HPCAs-0.4-900.

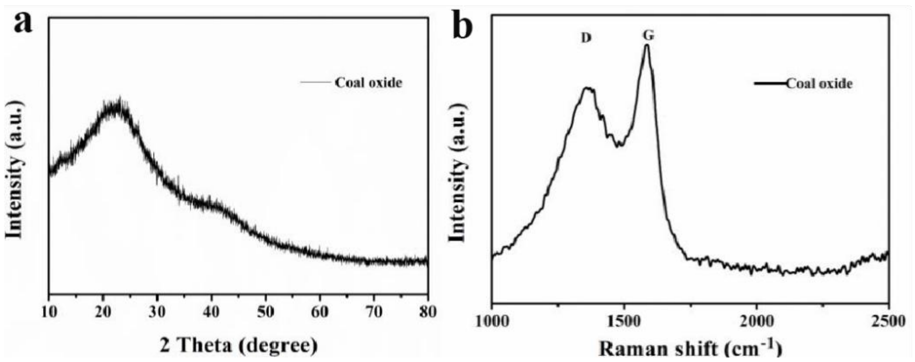

Figure S4. (a) XRD pattern and (b) Raman spectrum of coal oxide.

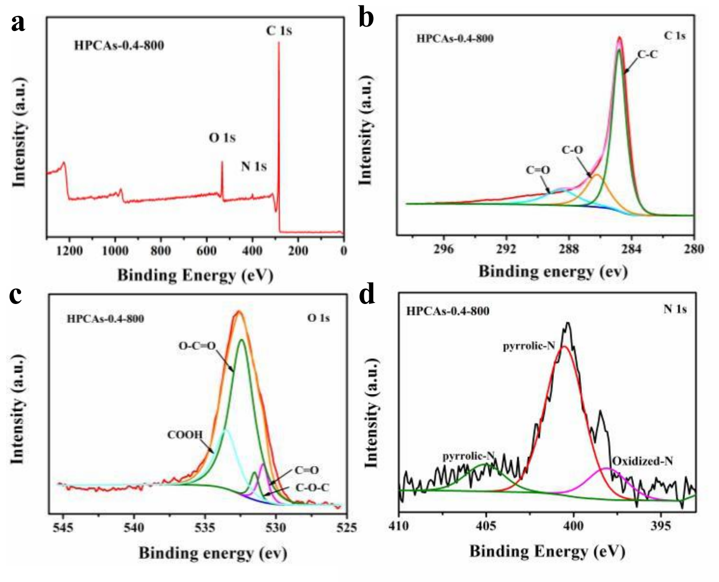

Figure S5. XPS spectra of HPCAs-0.4-800 samples (a) High resolution XPS spectra; (b) C1s;(c) O1s;(d) N1s.

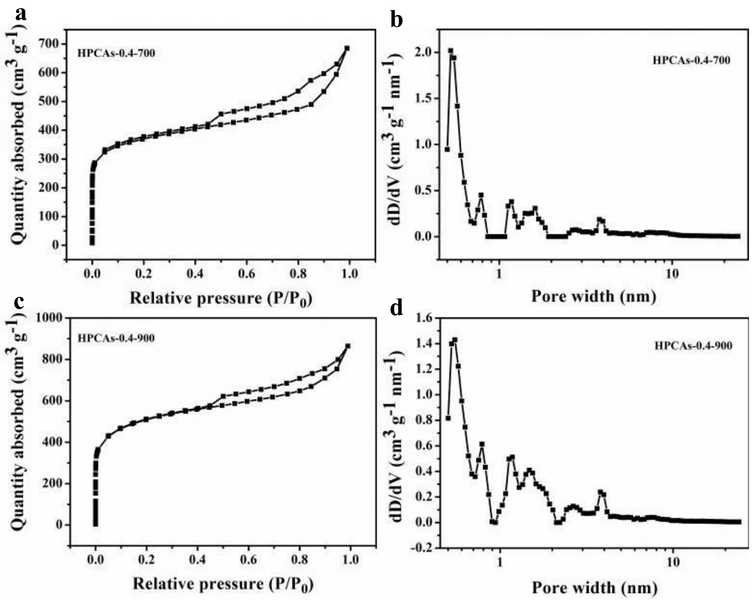

Figure S6. (a,c) N_2 adsorption-desorption isotherms; (b,d) and pore size distributions of HPCAs-0.4-700, HPCAs-0.4-900.

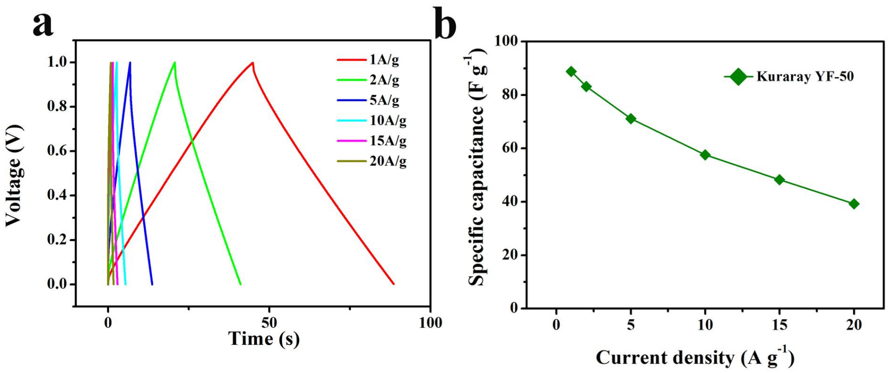

Figure S7. (a) GCD cures of the YF-50 at different current densities; (b) Specific capacitances of YF-50 at the current density of 0.5-20 A g^{-1}.

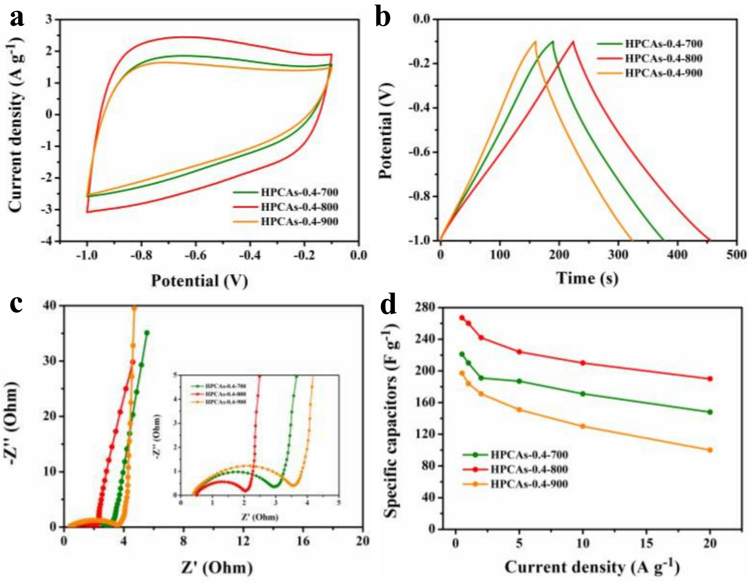

Figure S8. (a) CV curves at the scan rate of 50 mV s^{-1}; (b) GCD curves at the same current density at 1.0 A g^{-1} of the samples with different PVA content; (c) Electrochemical impedance spectra of HPCAs as Nyquist plots; (d) Specific capacitances of the samples with different temperature at the current density of 0.5-20.0 A g^{-1}.

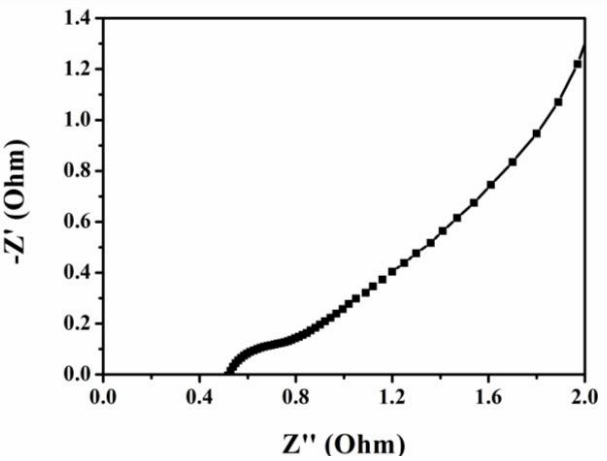

Figure S9. Electrochemical performances of the HPCAs-0.4-800//HPCAs-0.4-800 symmetric cell Nyquist plots.

Table S1. The elemental contents (atomic%) of the HPCAs-0.4-800 calculated from XPS data.

Sample	C (at.%)	O (at.%)	N (at.%)
HPCAs-0.4-800	91.13	7.27	1.6

Table S2. BET specific surface areas and porous structure of samples.

Sample	S_{BET}[a] (m^2 g^{-1})	V_{total}[b] (cm^{-3} g^{-1})	V_{meso}[c] (cm^{-3} g^{-1})	S_{micro}[d] (m^2 g^{-1})	D_{ap}[e] (nm)
HPCAs-0.4-700	971	1.34	0.67	526	5.5
HPCAs-0.4-800	1303	1.45	0.61	856	4.4
HPCAs-0.4-900	710	1.06	0.56	330	6.0
Pure oxidized coal-800	537	0.29	0.11	421	2.1
YP-50	1608	0.72	0.12	1526	2.3

[a]BET surface area. [b]The total pore volume at P/P_o=0.99, [c]The mesopore volume calculated using the BJH method based on the Kelvin equation. [d]Micropore surface area calculated using the V-t plot method. [e]Average pore size ($4V_t/S_{BET}$).

Table S3. Structural properties and electrochemical performances of carbon-based materials for supercapacitors.

Electrode materials	Specific Capacitance	Electrolyte	Current density	reference
Pomelo mesocarps nanosheets	245 F g^{-1}	6 M KOH	0.5 A g^{-1}	54
Beehive-like hierarchical nanoporous carbon	250 F g^{-1}	6 M KOH	1 A g^{-1}	43
Glucose-based aerogels	201 F g^{-1}	6 M KOH	1 A g^{-1}	14
Ordered mesoporous carbon/graphene aerogel	197 F g^{-1}	6 M KOH	1 A g^{-1}	55
Graphene aerogel	229 F g^{-1}	6 M KOH	1 A g^{-1}	56
Graphene nanoribbons	189 F g^{-1}	6 M KOH	0.1 A g^{-1}	10
Hollow carbon nanospheres	201.5 F g^{-1}	6 M KOH	5 mV s^{-1}	57
Coal-based carbon aerogels	260 F g^{-1}	6 M KOH	1 A g^{-1}	**This work**
